# Supplementary material for: Improving data sharing between acute hospitals in England: an overview of health record system distribution and retrospective observational analysis of inter-hospital transitions of care
Source: BMJ Open. 2019 Dec 5;9(12):e031637. doi: 10.1136/bmjopen-2019-031637 (PMC7008454; doi:10.1136/bmjopen-2019-031637)
Supplement: Author's manuscript [file bmjopen-2019-031637.draft_revisions.pdf]

PEER REVIEW HISTORY

BMJ Open publishes all reviews undertaken for accepted manuscripts. Reviewers are asked to complete a checklist review form (**http://bmjopen.bmj.com/site/about/resources/checklist.pdf**) and are provided with free text boxes to elaborate on their assessment. These free text comments are reproduced below.

ARTICLE DETAILS

**TITLE (PROVISIONAL)**	Please add the following note to production: Improving data sharing between acute hospitals in England: An overview of health record system distribution and retrospective observational analysis of inter-hospital transitions of care
**AUTHORS**	Warren, Leigh; Clarke, Jonathan; Arora, Sonal; Darzi, Ara

VERSION 1 – REVIEW

**REVIEWER**	Mark Patterson<br>University of Missouri-Kansas City, School of Pharmacy. Kansas City, MO. USA.
**REVIEW RETURNED**	15-Jul-2019

**GENERAL COMMENTS**	Organization and alignment of objectives and research outcomes<br><br>I am having difficulty aligning all the research objectives with the primary and secondary endpoints... First, the objectives stated in the abstract should mirror more closely those stated in the introduction. Second, the reader should be able to distinguish which outcomes go with which objective.<br><br>Example:<br>Objectives from Abstract:<br>1. To determine the frequency of transitions of care between acute hospital trusts and health record systems in the English NHS service<br>2. To identify targets for improvement healthcare data sharing and interoperability<br>Objectives from introduction:<br>1. To identify the distribution of health record systems in acute hospitals in NHS in England.<br>2. To determine the frequency of transitions of care between acute hospital trusts and health record systems.<br>3. To identify some of the barriers and facilitators to data sharing between NHS England acute hospitals.<br><br>Endpoints in abstract:<br>1. Frequency of transitions of care between different acute hospital trusts and health record systems.<br>2. The spatial distribution of health record systems in England.<br><br>Endpoints in methods:<br>1. Frequency and distribution of patient encounters with different health record systems used in NHS<br>2. Distribution of health record systems in England<br>3. Number of patients that attend more than one trust

	4. Regional distribution of areas that could most readily improve data sharing by better EHR system coordination<br><br>I would suggest mapping out the outcomes to each of your objectives as an exercise to ensure that each “outcome” has an objective. Second, I would suggest that when you refer to these outcomes in each of the sections of the paper, they are all phrased the same way, so the reader does not have to go back and navigate to figure out which outcome goes with each objective. Doing this mapping will help you also organize the presentation of the results, and the discussion.<br><br>Abstract:<br>I would refer to ‘distribution’ as “geographical distribution”. Also in the results, make sure you have reported the various groups of outcomes you mentioned in objectives. For example, I do not see results of the spatial map in Figure 2, which I think is a very important finding.<br><br>Introduction<br><br>Very well written Provides context and significance of the key problem.<br>Lines 55-59 pg. 4 – Excellent rationale for the project, I would try to integrate this into the abstract—if authors are allowed to provide ‘background’ sentence prior to the research objective.<br><br>Study aims last paragraph – I do like how authors provide the overall goal, integrated with the approach and the study objectives – but I would make sure the phrasing of the objectives are consistent between the abstract and the introduction. See overview comments above regarding alignment.<br><br>Methods:<br><br>How do the primary and secondary outcomes ‘align’ with the research objectives? Which go with which? If these are secondary outcomes, so you also have a ‘secondary objective’. See overview comments above.<br>I am confused about page 6 lines 19 to 32—Are these data on the health record vendor? Alternatively, data on the hospital systems use of multiple or singular health record systems? All of the above? How was this data collected, and how are these aligned with the study outcomes/endpoints?<br>I am confused about the ‘matrix” (pg. 7 lines 39 to 44) – Can authors provide example of the matrix, and why was it used? How does this data align with the study outcomes/endpoints? How were these used to calculate the primary and/or secondary outcomes?<br>Pg. 7 lines 50 to 60 – these particular calculations—are these also primary or secondary endpoints? May consider creating a separate table/figure that outlines all outcomes in study in one column, then a second column stating how each outcome was calculated.<br><br>Results<br><br>Pg. 9, lines 17 to 37 – I would suggest continuing to use the percentage that were provided in parentheses, similar to the style in lines 11 to 15. This change will help the reader get an immediate sense of the prevalence of each vendor system.

	Pg. 11, lines 12 to 24 – these results are important, and are not mentioned in the abstract. Consider adding these to the abstract as well.<br><br>Discussion<br><br>Well written first paragraph. I suggest that the concept in lines 37 to 41 (pg. 11) also be included in the abstract, if authors have room to provide context immediately before the research objectives. These concepts state well the overall rationale for the study.<br>Pg. 12 lines 3 to 11 – I would also add these results to the abstract as well.<br>No LSOA regional alignment results were reported in the abstract. I would suggest adding them to abstract as well.<br>Pg. 12 lines 30 to 45 – Effective discussion on stating the limitation. Authors do need to go a step further to explain how/why this limitation affects the observed findings. For example, does this bias the results in any way, why or why not? In other words, does this limitation affect internal validity? Furthermore, does this impact authors' ability to generalize results (i.e., external validity) to other health records systems? Discuss further.<br>Pg. 13 lines 19 to 11 – I agree the methodological approach in this study is useful for quality improvement – but how? Be more specific. For example, does the value of a particular metric/outcome signal to a policy maker to focus on a set of system flaws that prior to this study, could not be identified effectively? Elaborate more to make a more solid argument/case for advocating this methods approach for QI work in the area of interoperability<br>Pg. 13 lines 24 to 28 -- I really like this insight / comment about how interoperability will only become more critical and prevalent in the future as aging population develop diseases that require more and more specialists to coordinate care across the system. Very good.<br>Pg. 13 lines 30 to 44 – This discussion was important – to talk about the need for 'encouragement' to adopt interoperable systems. We have the same needs and issues in the USA, even in the presence of policies that provide financial rewards (or impose financial penalties) on hospitals not complying with the 'meaningful use criteria' set forth by the federal government around 2009 or so. Even with these financial incentives, USA still having issues reaching interoperability. I would be curious to see more discussion from authors on national strategies/policies that would help with "encouragement" -- in both the 1) adoption of health record systems, and better sharing; 2) the compatibility between vendors. On the latter, I am curious as to how policies (or the market?) could incentivize vendors to work together? Would this be possible? Alternatively, does the responsibility fall onto the hospitals to purchase software "patches" that help with interoperability? More discussion on these nuances would be enlightening and strengthen the discussion

**REVIEWER**	Simon Jones<br>NYU Langone Health, USA
**REVIEW RETURNED**	30-Aug-2019

**GENERAL COMMENTS**	This is an important paper which will add much to the understanding of interoperability in both the UK and in other countries. For the most part it is very clearly written. However, I dd

	find the section on LSOA analysis took some time and multiple readings to understand. Perhaps the authors could simplify it? The authors should be commended for such an intersting paper

VERSION 1 – AUTHOR RESPONSE

RESPONSES TO REVIEWER'S COMMENTS

Reviewer(s)' Comments to Author:

Reviewer: 1

Reviewer Name

Mark Patterson

Institution and Country

University of Missouri-Kansas City, School of Pharmacy. Kansas City, MO. USA.

Please state any competing interests or state 'None declared':
None declared

Please leave your comments for the authors below
Organization and alignment of objectives and research outcomes

I am having difficulty aligning all the research objectives with the primary and secondary endpoints... First, the objectives stated in the abstract should mirror more closely those stated in the introduction. Second, the reader should be able to distinguish which outcomes go with which objective.

Example:
Objectives from Abstract:
1. To determine the frequency of transitions of care between acute hospital trusts and health record systems in the English NHS service
2. To identify targets for improvement healthcare data sharing and interoperability
Objectives from introduction:
1. To identify the distribution of health record systems in acute hospitals in NHS in England.
2. To determine the frequency of transitions of care between acute hospital trusts and health record systems.
3. To identify some of the barriers and facilitators to data sharing between NHS England acute hospitals.

Endpoints in abstract:
1. Frequency of transitions of care between different acute hospital trusts and health record systems.
2. The spatial distribution of health record systems in England.

Endpoints in methods:
1. Frequency and distribution of patient encounters with different health record systems used in NHS
2. Distribution of health record systems in England
3. Number of patients that attend more than one trust

4. Regional distribution of areas that could most readily improve data sharing by better EHR system coordination

I would suggest mapping out the outcomes to each of your objectives as an exercise to ensure that each “outcome” has an objective. Second, I would suggest that when you refer to these outcomes in each of the sections of the paper, they are all phrased the same way, so the reader does not have to go back and navigate to figure out which outcome goes with each objective. Doing this mapping will help you also organize the presentation of the results, and the discussion.

We thank the reviewer for highlighting the need for greater clarity regarding the research objectives and study endpoints. In response to these recommendations we have mapped out the objectives and corresponding outcomes within the paper and rewritten/restructured these for clarity in the abstract, introduction and methods sections. We have also followed on with the same structure and language in the results and discussion sections to ensure consistency throughout the paper. We agree that this makes the paper clearer and easier for the reader to correlate objectives with outcomes.

Rather than placing a hierarchical (primary/secondary) restriction on the study objectives, and to simplify this issue, we have avoided the use of the terms ‘primary’ and ‘secondary’ in the paper and referred simply to study objectives. We have done our best to further clarify this approach and maintain consistency through the different sections of the paper but would welcome any further recommendations following review of these amendments.

Abstract:
I would refer to ‘distribution’ as “geographical distribution”. Also in the results, make sure you have reported the various groups of outcomes you mentioned in objectives. For example, I do not see results of the spatial map in Figure 2, which I think is a very important finding.

We thank the reviewer for their recommendation to clarify the meaning of the term ‘distribution’ in the paper. In response to this comment, and following discussion amongst the authors, we have referred to the distribution as ‘spatial distribution’ where relevant. We felt this clarified the term better and preferred the use of ‘spatial’ over ‘geographical’ when referring to distribution.

Introduction

Very well written Provides context and significance of the key problem.
Lines 55-59 pg. 4 – Excellent rationale for the project, I would try to integrate this into the abstract—if authors are allowed to provide ‘background’ sentence prior to the research objective.

We thank the reviewer for their supportive comments. Unfortunately, the structured abstract format stipulated by the journal commences with ‘objectives’ and does not allow a ‘background’ sentence to provide prior context. As described above, we have instead attempted to restructure and simplify the objectives for clarity.

Study aims last paragraph – I do like how authors provide the overall goal, integrated with the approach and the study objectives – but I would make sure the phrasing of the objectives are consistent between the abstract and the introduction. See overview comments above regarding alignment.

As discussed above, we have rephrased and restructured the objectives to ensure consistency between the abstract, introduction and methods sections.

Methods:

How do the primary and secondary outcomes 'align' with the research objectives? Which go with which? If these are secondary outcomes, so you also have a 'secondary objective'. See overview comments above.

As this research addresses several equally important outcomes that are relevant to policy, we have chosen to refer simply to 'outcomes' rather than a hierarchical 'primary' and 'secondary' outcome description (apart from where required in the abstract). As discussed above, we have made several changes to better align objectives and outcomes following these helpful suggestions.

I am confused about page 6 lines 19 to 32—Are these data on the health record vendor? Alternatively, data on the hospital systems use of multiple or singular health record systems? All of the above? How was this data collected, and how are these aligned with the study outcomes/endpoints?

In response to this comment/query, we have made some changes to this section of the methods. We have changed the title of this section to 'Hospital trust health record system usage'. We have also added some clarifying terms to this paragraph to indicate that this section refers to the data pertaining to health record type (e.g. paper/EHR) and vendor (if EHR). Regarding the query relating to the relevance to outcomes/endpoints, we have added the following line to this section: 'these data were used to calculate the frequency of use of health record systems.'.

I am confused about the 'matrix" (pg. 7 lines 39 to 44) – Can authors provide example of the matrix, and why was it used? How does this data align with the study outcomes/endpoints? How were these used to calculate the primary and/or secondary outcomes?

This section of the methods refers to the measurement of transition of care frequencies between each possible pair of trusts and health record systems. To clarify this paragraph we have removed the description of the 'matrix' (tables) and replaced this with a simplified description.

Pg. 7 lines 50 to 60 – these particular calculations—are these also primary or secondary endpoints? May consider creating a separate table/figure that outlines all outcomes in study in one column, then a second column stating how each outcome was calculated.

The calculations described in this section were used to generate the input for figure 2. The title for this section has been changed to 'Identifying the spatial distribution of transitions of care between health record systems' which we hope clarifies the rationale for this and relevance to the objective/endpoint as described above.

Results

Pg. 9, lines 17 to 37 – I would suggest continuing to use the percentage that were provided in parentheses, similar to the style in lines 11 to 15. This change will help the reader get an immediate sense of the prevalence of each vendor system.

As recommended by the reviewer, we have added further percentages where relevant in this section.

Pg. 11, lines 12 to 24 – these results are important, and are not mentioned in the abstract. Consider adding these to the abstract as well.

Our preference would also have been to include these results in the abstract, however unfortunately the word count for the structured abstract in BMJ Open is 300 words so this has not been possible.

Discussion

Well written first paragraph. I suggest that the concept in lines 37 to 41 (pg. 11) also be included in the abstract, if authors have room to provide context immediately before the research objectives.

Unfortunately, the structured abstract required by BMJ Open does not allow for a background to the study to be provided in the abstract.

These concepts state well the overall rationale for the study.
Pg. 12 lines 3 to 11 – I would also add these results to the abstract as well.

As above.

No LSOA regional alignment results were reported in the abstract. I would suggest adding them to abstract as well.

As above, unfortunately the word count limit in the abstract has restricted what we can include.

Pg. 12 lines 30 to 45 – Effective discussion on stating the limitation. Authors do need to go a step further to explain how/why this limitation affects the observed findings. For example, does this bias the results in any way, why or why not? In other words, does this limitation affect internal validity? Furthermore, does this impact authors' ability to generalize results (i.e., external validity) to other health records systems? Discuss further.

We thank the reviewer for considering this important section of the discussion and providing some further guidance on how to address some of these complexities inherent within research in this field. Following consideration of these comments we have added the following sentences to the discussion regarding the impact of these limitations on the internal and external validity of the study. "These limitations associated with the definition and dynamic nature of EHR usage are an unavoidable complexity of research in this field but did not significantly impact the main conclusions of this study. Clearer policy definitions of EHR usage and maintenance of a regularly updated national database of hospital health record systems would aid future analyses of contemporaneous patterns of care transitions between health record systems. Although the methods used in this study may translate to other international settings, similar limitations regarding the definitions of EHR usage and dynamic nature of health record systems in hospitals may exist."

Pg. 13 lines 19 to 11 – I agree the methodological approach in this study is useful for quality improvement – but how? Be more specific. For example, does the value of a particular metric/outcome signal to a policy maker to focus on a set of system flaws that prior to this study, could not be identified effectively? Elaborate more to make a more solid argument/case for advocating this methods approach for QI work in the area of interoperability

This is a helpful comment that we have used to guide the addition of comments in the discussion section. We have added sentences relating to interoperability quality improvement and the use of metrics, including the proportion of patients with consecutive encounters at trusts using different health record systems, to the discussion.

Pg. 13 lines 24 to 28 -- I really like this insight / comment about how interoperability will only become more critical and prevalent in the future as aging population develop diseases that require more and more specialists to coordinate care across the system. Very good.

Pg. 13 lines 30 to 44 – This discussion was important – to talk about the need for ‘encouragement’ to adopt interoperable systems. We have the same needs and issues in the USA, even in the presence of policies that provide financial rewards (or impose financial penalties) on hospitals not complying with the ‘meaningful use criteria’ set forth by the federal government around 2009 or so. Even with these financial incentives, USA still having issues reaching interoperability. I would be curious to see more discussion from authors on national strategies/policies that would help with “encouragement” -- in both the 1) adoption of health record systems, and better sharing; 2) the compatibility between vendors. On the latter, I am curious as to how policies (or the market?) could incentivize vendors to work together? Would this be possible? Alternatively, does the responsibility fall onto the hospitals to purchase software “patches” that help with interoperability? More discussion on these nuances would be enlightening and strengthen the discussion

We agree with the reviewer that these are incredibly interesting and important questions. As a primarily research paper, however, we have chosen not to comment specifically on policy questions beyond highlighting the need for encouragement to improve system alignment and interoperability in this article. Instead, we are planning further pieces with more of a policy focus, that draw on the conclusions from this research work. We hope that this research paper serves primarily to highlight the problems with the current ‘state of play’ in England that we, and others, can address in a different format. Many of these questions are difficult to answer and potentially controversial, and we did not want our opinions on these to impact on the research work presented here. We hope that the reviewer understands and agrees with these sentiments as we look forward to an ongoing discussion around these important policy issues with yourself and others going forward.

Once again, we would like to offer our sincere thanks for your helpful insights and comments regarding our paper. We feel that the paper is now stronger as a result and we look forward to hearing back from the journal in the near future regarding a final decision on publication.

Reviewer: 2

Reviewer Name

Simon Jones

Institution and Country

NYU Langone Health, USA

Please state any competing interests or state ‘None declared’:
None

Please leave your comments for the authors below

This is an important paper which will add much to the understanding of interoperability in both the UK and in other countries. For the most part it is very clearly written.

However, I dd find the section on LSOA analysis took some time and multiple readings to understand. Perhaps the authors could simplify it?

We thank the reviewer for their supportive comments regarding our paper. In response to this recommendation we have amended and simplified this paragraph regarding the LSOA/spatial analysis in the methods section.

The authors should be commended for such an interesting paper

VERSION 2 – REVIEW

**REVIEWER**	Simon Jones<br>NYU Medical School
**REVIEW RETURNED**	11-Nov-2019

**GENERAL COMMENTS**	The authors have improved many aspects of this excellent paper. I very much hope that it is accepted.
